# Endothelial Dysfunction Driven by Hypoxia—The Influence of Oxygen Deficiency on NO Bioavailability

**DOI:** 10.3390/biom11070982

**Published:** 2021-07-03

**Authors:** Anna Janaszak-Jasiecka, Anna Siekierzycka, Agata Płoska, Iwona T. Dobrucki, Leszek Kalinowski

**Affiliations:** 1 Department of Medical Laboratory Diagnostics—Fahrenheit Biobank BBMRI.pl, Medical University of Gdansk, 80-211 Gdansk, Poland; anna.janaszak-jasiecka@gumed.edu.pl (A.J.-J.); anna.siekierzycka@gumed.edu.pl (A.S.); agata.ploska@gumed.edu.pl (A.P.); 2 Biobanking and Biomolecular Resources Research Infrastructure Poland (BBMRI.pl), 80-211 Gdansk, Poland; 3 Laboratory of Trace Elements Neurobiology, Institute of Pharmacology, Polish Academy of Sciences, 31-343 Krakow, Poland; 4 University of Illinois at Urbana-Champaign Beckman Institute for Advanced Science and Technology, 405 N Mathews Ave, MC-251, Urbana, IL 61801, USA; dobrucka@illinois.edu; 5 Department of Bioengineering, University of Illinois at Urbana-Champaign, Urbana, IL 61801, USA; 6 BioTechMed Centre, Department of Mechanics of Materials and Structures, Gdansk University of Technology, 80-233 Gdansk, Poland

**Keywords:** nitric oxide, eNOS, eNOS uncoupling, tetrahydrobiopterin, ADMA, hypoxia, cardiovascular diseases

## Abstract

Cardiovascular diseases (CVDs) are the leading cause of death worldwide. The initial stage of CVDs is characterized by endothelial dysfunction, defined as the limited bioavailability of nitric oxide (NO). Thus, any factors that interfere with the synthesis or metabolism of NO in endothelial cells are involved in CVD pathogenesis. It is well established that hypoxia is both the triggering factor as well as the accompanying factor in cardiovascular disease, and diminished tissue oxygen levels have been reported to influence endothelial NO bioavailability. In endothelial cells, NO is produced by endothelial nitric oxide synthase (eNOS) from L-Arg, with tetrahydrobiopterin (BH_4_) as an essential cofactor. Here, we discuss the mechanisms by which hypoxia affects NO bioavailability, including regulation of eNOS expression and activity. What is particularly important is the fact that hypoxia contributes to the depletion of cofactor BH_4_ and deficiency of substrate L-Arg, and thus elicits eNOS uncoupling—a state in which the enzyme produces superoxide instead of NO. eNOS uncoupling and the resulting oxidative stress is the major driver of endothelial dysfunction and atherogenesis. Moreover, hypoxia induces impairment in mitochondrial respiration and endothelial cell activation; thus, oxidative stress and inflammation, along with the hypoxic response, contribute to the development of endothelial dysfunction.

## 1. Introduction

The endothelium plays a pivotal role in maintaining cardiovascular homeostasis. Endothelial cells produce and release a subset of substances that regulate vascular tone and blood flow, hemostasis, vascular permeability, angiogenesis, and immunity. Endothelium-dependent regulation of vascular tone is dependent on several vasoactive factors. The most important vasodilators include nitric oxide (NO), prostacyclin (PGI2), and endothelium-derived hyperpolarizing factor (EDHF), whereas vasoconstrictive factors are mainly endothelin-1 (ET-1), thromboxane (TXA2), and angiotensin II [1,2]. Among these substances, NO is probably the key molecule, as its antiatherogenic properties ensure proper vascular physiology [3]. NO dilates blood vessels, inhibits vascular smooth muscle cell proliferation, platelet aggregation, and leukocyte adhesion; thus, adequate NO bioavailability determines vascular health. The impairment of the homeostatic endothelial cell functions leads to endothelial dysfunction, defined mainly as decreased NO bioavailability. Consequently, reduced vasodilation and accompanying pathologies contribute to the pathomechanism of atherosclerosis, which in turn leads to severe cardiovascular complications [4,5].

The cardiovascular system delivers oxygen and nutrients to all tissues of the body. Changes in tissue oxygen availability affect normal physiology and are often involved in the development of pathological conditions; thus, the optimal oxygen concentration is critical to maintaining homeostasis. Low blood oxygen (hypoxemia) may result from reduced inspired oxygen tension (e.g., high altitude sickness), respiratory system pathologies, e.g., asthma, chronic obstructive pulmonary disease (COPD), pneumonia, acute respiratory distress syndrome (ARDS)—also in the course of COVID-19, or other pathologies (e.g., anemia, sleep apnea, heart failure) [6,7,8,9]. Hypoxemia is detected by carotid bodies and results in rapid systemic response: increased breathing rate and heart rate, dilation of peripheral blood vessels, and constriction of lung vessels—all aimed at restoration of proper blood oxygenation [10]. Hypoxemia most often leads to hypoxia, a condition in which the oxygen level is insufficient to cover the needs of a particular organ or tissue. Furthermore, local hypoxia caused by impaired oxygen distribution from blood to tissues accompanies many pathological states, such as stroke, myocardial infarction, atherosclerosis, and cancer, and contributes to the exacerbation of adverse lesions [11,12].

Generally, hypoxia can be divided according to its duration into acute or chronic, or according to its nature into persistent or intermittent. For example, chronic lung diseases result in persistent hypoxia, while obstructive sleep apnea (OSA) is associated with intermittent hypoxia, consisting of cycles of hypoxia and reoxygenation [13]. The hypoxic response differs slightly depending on the nature of the hypoxia [14]. In the cellular models of intermittent hypoxia, the augmented proangiogenic and proinflammatory phenotype was observed [14]. Moreover, severe hypoxia followed by reoxygenation may cause ischemia-reperfusion injury, the phenomenon of cellular injury observed after myocardial ischemia, stroke, or organ transplantation resulting from increased reactive oxygen species (ROS) generation [15]. Intermittent hypoxia associated with OSA can also lead to ischemia-reperfusion injury, which is recognized as a major contributor to the pathogenesis of OSA comorbidities via increased ROS production [16,17].

At the cellular level, the hypoxic response triggers molecular mechanisms of transcriptional reprogramming, dependent mainly on hypoxia inducible factors (HIFs). HIFs are heterodimers composed of two subunits—constitutive β subunit, and one of three oxygen-dependent α subunits HIF-1α, HIF-2α or HIF-3α [18]. In normoxia, HIF-α is hydroxylated by specific prolyl hydroxylases (PHDs), using O_2_ as a substrate. Hydroxylation of HIF-α enables its interaction with pVHL (von Hippel–Lindau protein) and subsequent recruitment of ubiquitin ligase, leading to proteasomal degradation of HIF-α. Under hypoxic conditions, PHDs activity is inhibited, HIF-α consequently accumulates, translocates to the nucleus, and together with HIF-β subunit forms an active transcription factor (HIF-1, HIF2 or HIF-3) [19]. Therefore, the hypoxic response at the molecular level relies on the induction of transcription of a specific set of genes characterized by the presence of HIF responsive elements (HREs) within their regulatory regions. The HIF-driven transcriptional response allows adaptation to low oxygen levels or counteracts the effects of hypoxia, i.a. by inducing the expression of genes promoting erythropoiesis, angiogenesis, and glycolysis; however, it also contributes to the initiation of pathological processes [20,21]. HIF-1α and HIF-2α share 48% amino-acid sequence identity and structural similarity; nonetheless, they are differentially regulated [18]. HIF-1α is expressed in nearly all cell types, whereas HIF-2α expression is limited to certain tissues, including endothelium, lungs, kidneys, brain, liver, and heart. Moreover, HIF-1α and HIF-2α are often overexpressed in cancer tissue [22]. HIF-3α differs from HIF-1α and HIF-2α in protein structure, and much less is known about its role and target genes [23].

All three HIF isoforms are expressed in the endothelium, but the time of induction and the role of individual HIFs are different. HIF-1α accumulates in the initial phase of hypoxia, while the accumulation of HIF-2α follows prolonged, chronic hypoxia when HIF-1α decreases [18,24]. Further persistent oxygen depletion also leads to HIF-3α accumulation [25]. Individual HIF isoforms stimulate the expression of distinct sets of target genes, which partially overlap. HIF-1 is responsible for the induction of genes associated with the glycolytic pathway (phosphofructokinase (PFK), lactate dehydrogenase (LDHA), pH regulation (monocarboxylate transporter 4 (MCT4) and carbonic anhydrase 9 (CA-IX)), and apoptosis induction (BCL2/adenovirus E1B 19 kDa-interacting protein 3 (BNIP3) and BCL2/adenovirus E1B 19 kDa-interacting protein 3-like (BNIP3L/NIX)) [26]. HIF-2 drives the induction of angiogenesis by stimulating the expression of erythropoietin (EPO) and matrix metalloproteinases (MMP) [26,27,28]. HIF-1 and HIF-2 share common target genes (e.g., vascular endothelial growth factor A (VEGF), glucose transporter 1 (GLUT1)). Moreover, HIFs functions can replace each other under specific conditions, e.g., in the absence of HIF-1, HIF-2 can induce genes normally dependent on HIF-1 and vice versa [18].

The endothelium is the first layer of cells to contact with blood and is the first to be exposed to any changes in oxygen levels. Hypoxia and HIF signaling have a significant impact on endothelial function and biology, especially by induction of genes related to angiogenesis (VEGF) and glycolysis (glycolytic enzymes, glucose transporters) [29]. Importantly, hypoxia also affects NO generation by endothelial cells through modulating the expression and activity of endothelial nitric oxide synthase (eNOS). This review focuses on the hypoxic dysregulation of the eNOS pathway and highlights the mechanisms by which hypoxia contributes to the development of endothelial dysfunction.

## 2. eNOS and Its Regulation

NO is produced from L-arginine (L-Arg) and oxygen (O_2_) in a reaction catalyzed by nitric oxide synthase (NOS). Three isoforms of NOS have been described in humans: neuronal (nNOS) localized mainly in the nervous system cells, inducible NOS (iNOS) which expression is induced in various cell types by proinflammatory cytokines, and endothelial NOS (eNOS), expressed almost exclusively in endothelial cells [30]. This tissue-specific expression of eNOS was shown to be controlled through epigenetic mechanisms including specific DNA methylation patterns and post-translational histone modifications [31,32]. Different NOS isoforms generate NO at different rates, and NO concentration is a key determinant of its function. iNOS is the most potent NO donor, and high NO concentrations (e.g., produced by activated macrophages) have a cytostatic and cytotoxic effect. eNOS, in turn, generates the lowest NO levels capable of activating soluble guanylate cyclase (sGC) to generate the second messenger cGMP resulting in i.a. vasorelaxation and inhibition of platelet aggregation, thus preventing atherogenesis [33,34]. Therefore, endothelium-derived NO plays a pivotal role in maintaining vascular homeostasis, and the proper eNOS activity is critical for vascular health [35].

Functional eNOS is a homodimer consisting of two identical monomers of 134 kDa. The C-terminal reductase domain of one monomer is linked to the N-terminal oxygenase domain of the other monomer. The reductase domain possesses binding sites for NADPH (nicotinamide adenine dinucleotide phosphate), FMN (flavin mononucleotide), and FAD (flavin adenine dinucleotide), whereas the oxygenase domain binds the heme group, zinc, the cofactor tetrahydrobiopterin (BH_4_), and the substrate L-Arg. eNOS catalyzes the flavin-mediated electron transfer from C-terminal-bound NADPH of one monomer to the N-terminal oxygenase domain of the second monomer to convert L-arginine to NO and L-citrulline [36]. Moreover, the BH_4_ cofactor is essential for optimal eNOS activity, facilitating the transfer of electrons for the oxidation of L-Arg between the C- and N-terminal domains [37].

Although eNOS is considered as constitutively expressed in ECs, its expression level is adjusted by various transcriptional and posttranscriptional mechanisms in response to physiological or pathological stimuli, such as cell growth, shear stress, oxidative stress and inflammation [38]. Additionally, the enzyme activity is subjected to complex regulation through its posttranslational modifications such as phosphorylation, acetylation, fatty-acid acylation, S-nitrosylation, and protein-protein interactions [39,40]. Palmitoylation and myristoylation of eNOS enable its localization to the plasmalemmal caveolae, where the enzyme is sequestered in its inactive state due to the interaction with caveolin-1 [41]. eNOS activity is strictly related to intracellular calcium concentration. When intracellular calcium levels increase, calcium-activated calmodulin binds eNOS, disrupts its interaction with caveolin, and stimulates NO synthesis [39]. Moreover, eNOS activity is also regulated by its phosphorylation status, dependent on the activity of protein kinases (mainly PKA, Akt, and AMPK) and phosphatases. Phosphorylation at Ser1177, Ser633 and Ser615 stimulate eNOS, whereas phosphorylation at Thr495 and Ser114 inhibits it [42]. Activation of eNOS may occur in response to diverse stimuli, including shear stress, acetylcholine, bradykinin, or hormones, acting through changes in eNOS interactions or its phosphorylation status [39]. Blood flow-induced laminar shear stress is thought to be crucial for physiological eNOS expression and activity, and conversely, disturbed or oscillatory flows near arterial bifurcations are associated with atherosclerotic changes [43]. Laminar shear stress stimulates influx of calcium, calmodulin-eNOS binding, as well as calcium-independent eNOS phosphorylation at Ser1177, all resulting in the proper NO generation [44]. On the other hand, eNOS activity can be stimulated by receptor-dependent agonists (acetylcholine, bradykinin) acting through specific receptors that activate G-protein-dependent signaling pathways, leading to the release of intracellular calcium, and eNOS activation [36].

Endothelial NO production is also influenced by hormones that rapidly affect eNOS activity by altering its phosphorylation or modulate the amount of eNOS protein [45]. Insulin, thyroid hormones, and estrogen have been shown to increase eNOS expression as well as Akt-dependent phosphorylation of the enzyme at Ser1177 [46,47,48,49,50,51]. Accordingly, insulin resistance and hypothyroidism are associated with reduced eNOS activity and increased risk of CVD [52,53]. Importantly, the impact of hormones on eNOS explains in part the differences in CVD incidence between men and women. Estrogen is thought to significantly inhibit the development of atherosclerosis through stimulation of eNOS expression and its activity [50,54,55]. High estrogen levels in women reduce the risk of cardiovascular disease, but the risk dramatically increases after menopause [56]. The role of estrogen in the modulation of eNOS has been reviewed in detail elsewhere [57].The activity of eNOS is also stimulated by its interaction with the Hsp90 chaperone. HSP90 promotes the dissociation of eNOS from caveolin-1; furthermore, it protects eNOS against proteolysis and increases the rate of Akt-dependent eNOS phosphorylation, collectively contributing to increased NO production [39,58]. Although eNOS also interacts with several other proteins, these are beyond the scope of the review and are not mentioned here.

### eNOS Uncoupling

Physiologically, eNOS catalyzes the interdomain electron transfer from NADPH to L-Arg to produce NO, so electron transfer is “coupled” to NO synthesis. However, under various pathological conditions, eNOS becomes “uncoupled”—electrons leak from the transport chain in the reductase domain and are transferred to molecular oxygen to yield superoxide (O_2_^−^) instead of NO. Thus, so-called eNOS uncoupling changes eNOS from beneficial, NO producing enzyme to harmful O_2_^−^ source. Moreover, O_2_^−^ reacts with NO, yielding peroxynitrite (ONOO^−^), a highly reactive oxidant, which rapidly oxidizes BH_4_. As a result, overall NO bioavailability is reduced, and eNOS contributes to oxidative stress, being a generator of O_2_^−^ and ONOO^−^ [59]. Several mechanisms are implicated in eNOS uncoupling, including deficiency of cofactor BH_4_, depletion of substrate L-Arg, and accumulation of asymmetric dimethylarginine (ADMA) which is a competitive inhibitor of eNOS [60]. Importantly, eNOS uncoupling is implicated in the pathophysiology of cardiovascular diseases, and the accompanying oxidative stress is considered the major culprit [61,62,63].

## 3. Hypoxia and Cardiovascular Diseases

While the hypoxic response allows cells and tissues to maintain homeostasis, it also has pathological effects that play a pivotal role in the pathogenesis of cancer, metabolic disease, and, notably, cardiovascular disease, which is the subject of this review. Hypoxia appears to be a common feature of many CVDs, and lack of oxygen can both trigger the development of CVD as well as accompany this disease, thus exacerbating adverse changes and contributing to the disease progression [64].

Respiratory disorders such as chronic obstructive pulmonary disease (COPD) or obstructive sleep apnea (OSA), as well as long-term exposure to high altitudes results in hypoxemia. Insufficient blood oxygenation, in turn, evokes hypoxic pulmonary vasoconstriction, which, if it persists, eventually leads to the development of pulmonary hypertension (PH) [65,66]. It was demonstrated that the HIF pathway is involved in the pathogenesis of PH. Mice partially deficient for HIF-1α or HIF-2α have been shown to be protected from hypoxia-induced PH [67,68]. Interestingly, the pathophysiology of PH has been linked to the disruption of the NO pathway [69]. Intrapulmonary NO levels were reported to be decreased in PH patients [70], implicating that endothelial dysfunction is involved in the pathogenesis of PH. Similarly, the main mechanism implicated in OSA pathophysiology is reduced NO production and endothelial dysfunction [71]. OSA is characterized by chronic intermittent hypoxia and is considered an independent risk factor for CVD. Serum nitrites and nitrates as derivatives of circulating nitric oxide were reported to be reduced in OSA patients [72,73]. Consequently, endothelium-dependent vasodilation is impaired in these subjects, which favors the development of CVD [74]. Hence, hypoxia may contribute to the development of endothelial dysfunction.

On the other hand, cardiovascular diseases are often accompanied by inadequate oxygen supply. Hypoxic areas are a typical element of atherosclerotic lesions, as the thickness of the plaque limits oxygen diffusion and, additionally, a large portion of available oxygen is consumed by accumulated foam cells [75]. The presence of hypoxic regions was demonstrated in the arterial walls of rabbits with experimentally induced atherosclerosis [76]. Hypoxia was also detected in human atherosclerotic carotid arteries, where it has been demonstrated to activate HIFs, and through the action of VEGF, stimulate intraplaque angiogenesis [77]. Neovascularization has been associated with plaque growth, instability, and rupture and, therefore, hypoxia is implicated in the progression of atherosclerosis [78].

Diminished NO production and bioavailability are often involved in the pathogenesis of hypoxia-related diseases and cardiovascular complications. Here, we focus on the cellular aspects of hypoxia and its influence on the elements of nitric oxide-producing machinery. Decreased NO bioavailability may result from (i) decreased expression or activity of eNOS (ii) uncoupling of eNOS (iii) oxidative scavenging of NO by superoxide, leading to the formation of peroxynitrite (ONOO^−^).

The influence of hypoxia on eNOS uncoupling seems to be particularly important since uncoupled eNOS is a source of harmful radicals: superoxide and peroxynitrite, and the resulting oxidative stress underlies the pathophysiology of atherosclerosis. Below, we will discuss molecular mechanisms by which hypoxia contributes to the reduction in the synthesis and bioavailability of NO in the pathophysiology of cardiovascular diseases.

## 4. Influence of Hypoxia on eNOS Expression

eNOS expression and activity are regulated at the transcriptional, posttranscriptional and posttranslational levels [38]. Any disturbance of this complex regulation is reflected by changes in NO bioavailability and affects cardiovascular health. Various stimuli, such as oxidative stress, inflammation, and also hypoxia, affect eNOS expression and activity and thus contribute to the pathogenesis of cardiovascular diseases. Inadequate oxygen supply is a well-confirmed modulator of eNOS; however, research findings in this field are somewhat inconsistent. Hypoxic regulation of eNOS expression is complex and ambiguous, dependent on the species (human vs. rodents and others), endothelial heterogeneity across distinct vascular beds, experimental model (in vitro cell culture or animal studies), or the stage of development. eNOS was shown to be downregulated by hypoxia in vitro, in cultured human umbilical vein endothelial cells (HUVECs) [79,80,81,82,83], human coronary artery endothelial cells (HCAECs) [84], bovine pulmonary artery endothelial cells (PAECs) [85], human saphenous vein endothelial cells [86], as well as in vivo in the lungs of patients with pulmonary hypertension [87] or in aortas and mesenteric arteries of mice exposed to chronic intermittent hypoxia [88]. Interestingly, the effect of hypoxia on eNOS may vary depending on whether it is an arterial or a venous endothelium. In contrast to HUVECs, in human umbilical artery endothelial cells (HUAECs), hypoxia upregulated eNOS expression in vitro [89]. Similar regularity was observed in vivo, as eNOS was downregulated in HUVECs and upregulated in HUAECs collected from pregnancies affected by hypoxia (fetal growth restriction, FGR) [89,90,91]. eNOS upregulation was also observed in pulmonary endothelium of mice or rats exposed to hypoxia [92,93], and in vitro, in hypoxic porcine aortic endothelial cells [94]. Chronic hypoxia was also shown to upregulate eNOS expression in the endothelium from the uterine of pregnant sheep, but not in their femoral or renal arteries, nor in the uterine of non-pregnant sheep [95]. Some studies, in turn, show that hypoxia does not change the expression of eNOS but affects its enzymatic activity [96,97]. Despite these discrepancies, most studies seem to indicate a decreased expression of eNOS in hypoxia; however, one should keep in mind the overrepresentation of some research models (e.g., popular HUVEC cells). Since this review concerns the role of hypoxia in endothelial dysfunction, we will focus here on the mechanisms leading to decreased expression or activity of eNOS. Still, it should be remembered that it is not always the case.

**Figure 1:**
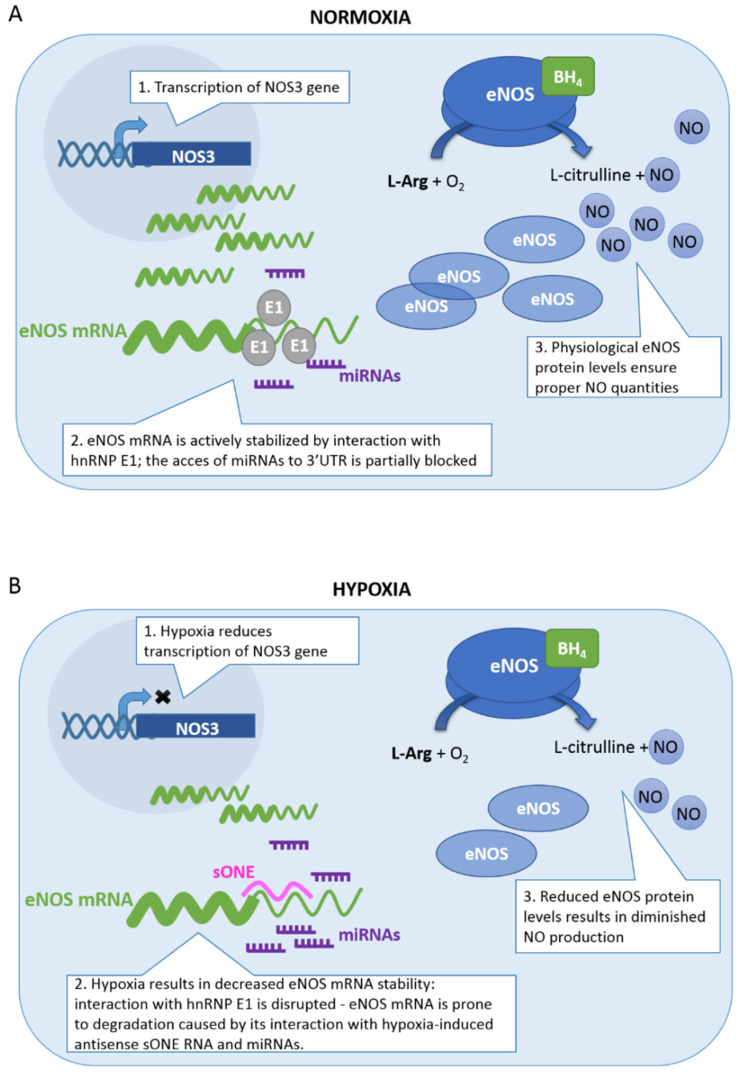
Regulation of eNOS expression in normoxia (**A**) and hypoxia (**B**). Hypoxic downregulation of eNOS is due to decreased transcription and reduced mRNA stability. In hypoxic ECs, eNOS mRNA is not stabilized by hnRNP E1, and its 3′-UTR interacts with hypoxia-induced antisense sONE transcript, as well as with miRNAs, resulting in increased eNOS mRNA degradation.

Several different mechanisms involved in eNOS downregulation have been described. It has been shown that hypoxia significantly reduces both the transcription of eNOS and stability of eNOS mRNA in human umbilical vein endothelial cells and bovine pulmonary artery endothelial cells (HUVECs and bovine PAECs), which results in reduced nitric oxide production [81,85]. Hypoxia may affect the DNA methylation status [98]. The CpG site located at position -171 in the promoter region of eNOS was reported to be hypermethylated in OSA pediatric patients, leading to reduced expression of eNOS [99]. Altered methylation pattern was also responsible for changed eNOS expression in HUVECs and HUAECs collected from FGR fetuses [90]. Fish et al. [79], in the study on hypoxic HUVEC, showed that the repression of eNOS transcription might be mediated through epigenetic regulation by histone modification and eviction. In turn, reduced stability of eNOS mRNA was attributed to its interaction with natural antisense sONE transcript. sONE, the long noncoding RNA (lncRNA), is complementary to eNOS mRNA in a region spanning the fragment of its coding sequence and 3′-UTR. Being expressed in endothelial cells at very low levels under normoxia, sONE is strongly upregulated by hypoxia, leading to eNOS message destabilization and reduction in eNOS protein level. In parallel, upregulation of sONE was also demonstrated in vivo in aortas of hypoxic rats [80]. Moreover, decreased eNOS mRNA stability may also result from its interaction with microRNAs [100]. As we previously demonstrated, hypoxia-induced miR-200b contributed to the reduction in eNOS expression and diminished NO release in hypoxic HUVECs [82]. Additionally, in normoxia, eNOS mRNA was reported to be actively stabilized by its interaction with heterogeneous nuclear ribonucleoprotein E1 (hnRNP E1) [83]. Hypoxia disrupts this interaction, making eNOS mRNA susceptible to destabilization with antisense RNA or miRNAs (Figure 1) [83]. Thus, microRNAs, which normally do not have access to the 3’-UTR, may interact with the 3’-UTR during hypoxia and negatively regulate eNOS expression. Even though the expression of these miRNAs is not upregulated by hypoxia, their functionality could be increased under hypoxic conditions due to the extended accessibility of eNOS mRNA. Several miRNAs that are not directly related to hypoxia have been implicated in eNOS regulation, including miR-155, miR-222/221, miR-24, and miR-765 [83,101,102,103]. Moreover, decreased eNOS mRNA stability in human endothelial cells has been attributed to yet another mechanism, dependent on the reorganization of the actin cytoskeleton via hypoxia-induced Rho kinase [86]. Of note, Rho kinase can also suppress eNOS activity through direct phosphorylation of eNOS at Thr495 [104].

Downregulation of eNOS by insufficient oxygen supply was observed in vivo as well. Analysis of aortas from rats exposed to hypoxia revealed a dramatic reduction in eNOS protein and mRNA and impairment of endothelium-dependent vascular relaxation, as compared to normoxic control [105]. Reduced expression of eNOS was observed in hypoxia-related diseases, i.e., in the lungs of patients with pulmonary hypertension [87] and in endothelial cells of OSA patients [106]. Diminished NO production is well documented and serves as a partial explanation of the pathogenesis of cardiovascular diseases, but on the other hand, several studies indicate that both expression and/or activity of eNOS may be upregulated by hypoxia. Induction of eNOS transcription was reported to be responsible for eNOS upregulation in hypoxic bovine aortic endothelial cells (BAECs); however, elevated eNOS level was not accompanied by its increased activity [107]. Similar results were obtained from in vivo animal studies—eNOS has been found to be upregulated by chronic hypoxia in rat lung tissue [105,108,109]. As mentioned above, these discrepancies in hypoxic regulation of eNOS expression may result from the use of different research models and different experimental approaches.

## 5. eNOS Activity in Hypoxia

In addition to regulating the amount of eNOS protein, hypoxia can also affect the enzymatic activity of eNOS by modulating its post-translational modifications. Hypoxia has been shown to alter eNOS phosphorylation status: eNOS Ser1177 phosphorylation and Akt kinase expression and phosphorylation were reduced in hypoxic HUVECs, whereas Thr495 phosphorylation was increased [110]. Increased eNOS Thr495 phosphorylation has also been observed in the lungs of patients with pulmonary hypertension [111]. Hypoxic inhibition of eNOS activity may be due to changes in protein–protein interactions. Oxygen deprivation has been reported to decrease HSP90 expression in porcine PAECs, thereby reducing the amount of HSP90 interacting with eNOS, leading to decreased eNOS activity [96]. In another study, decreased eNOS activity in the pulmonary arteries of hypoxic rats was attributed to attenuation of Ser1177 phosphorylation and alterations in eNOS interactions with caveolin and calmodulin [112].

## 6. eNOS Uncoupling Elicited by Hypoxia

eNOS uncoupling is considered the major cause of endothelial dysfunction observed in CVD. When eNOS itself becomes a source of superoxide and peroxynitrite anions, and NO is quenched, oxidative stress augments, leading to endothelial dysfunction and atherogenesis. Hypoxia can induce eNOS uncoupling by altering the availability of cofactor BH_4_ and substrate L-Arg, and the mechanisms contributing to these disturbances are discussed below.

### 6.1. BH_4_/BH_2_ Ratio

Tetrahydrobiopterin (BH_4_) is an essential cofactor for eNOS. Its cellular availability is an outcome of the de novo biosynthesis pathway, loss of BH_4_ by oxidation to dihydrobiopterin (BH_2_), and regeneration of the reduced form through the salvage pathway [113]. BH_4_ is synthesized de novo from GTP, with GTP cyclohydrolase I (GTPCH1) as the first and rate-limiting enzyme in this pathway [114]. Under oxidative stress conditions, BH_4_ is easily oxidized to BH_2_ by O_2_^−^ and uncoupled eNOS-derived ONOO^−^. The resulting BH_4_ deficiency is probably the main cause of eNOS uncoupling. BH_4_ can be regenerated from its oxidized form BH_2_ via the salvage pathway by dihydrofolate reductase (DHFR) [37]. Cardiovascular diseases are associated with oxidative stress, oxidation of BH_4_ to BH_2_, and eNOS uncoupling, implicating that the salvage pathway seems to have particular importance in their pathogenesis [115]. Indeed, DHFR has been suggested to play a critical role in regulating the BH_4_/BH_2_ ratio and eNOS coupling and activity in endothelial cells [116,117].

Hypoxia was reported to affect DHFR expression. As reported by Chalupsky et al. [118], the BH_4_/BH_2_ ratio and NO level were diminished in hypoxic human pulmonary artery endothelial cells (HPAECs) and in pulmonary arteries from mice exposed to hypoxia, due to reduced DHFR expression. Interestingly, hypoxia had no effect on the total amount of BH_4_ and BH_2_ but only changed their ratio, indicating that the de novo pathway is not affected by hypoxia, in contrast to the salvage pathway. Inhibition of DHFR expression by hypoxia hindered BH_4_ regeneration, resulting in eNOS uncoupling. Thus, downregulation of DHFR by hypoxia has been proposed to contribute to the pathogenesis of pulmonary hypertension. In the hypoxia-induced pulmonary hypertension rat model, BH_4_ levels were decreased, and exogenous BH_4_ supplementation augmented lung eNOS activity and reduced superoxide production [119,120].

### 6.2. L-Arg/ADMA Ratio

Hypoxia can affect the availability of L-Arg, the substrate for NO generation by eNOS. Intracellular L-Arg concentration depends on its dietary intake, whole-body protein turnover, endogenous synthesis, cellular uptake, and metabolism, with the major fraction of plasma L-Arg coming from protein breakdown [121,122].

Under physiological conditions, the intracellular L-Arg levels far exceed the Km of NO synthase, and eNOS is theoretically saturated with the substrate [123]. However, NO formation is dependent on extracellular L-Arg concentrations, a phenomenon known as the ‘L-arginine paradox’ [124]. In this context, the actual uptake of L-Arg by ECs regulates eNOS activity and emphasizes the role and efficiency of the L-Arg transporter. In endothelial cells, L-Arg is taken up mainly via the cationic amino acid transporter CAT-1, belonging to the system y+ carrier [125,126]. Interestingly, CAT-1 was reported to co-localize and interact with eNOS in plasma membrane caveolae [127]. This mutual proximity would facilitate the direct delivery of L-Arg to eNOS, further emphasizing the role of CAT-1 in the regulation of eNOS efficiency. Thus, factors that alter the efficiency or expression of the CAT-1 transporter would affect the synthesis of nitric oxide. There are only a few reports on hypoxic regulation of CAT-1 activity; nevertheless, they are consistent: hypoxia negatively regulates L-Arg uptake by ECs. Inadequate oxygen supply was demonstrated to inhibit L-Arg uptake as well as its intracellular content in porcine PAECs [128,129]. Consistently, overexpression of CAT-1 in hypoxic human pulmonary microvascular endothelial cells (PMVECs) increased NO production [130].

Once taken up by an endothelial cell, L-Arg can be metabolized by eNOS to generate NO, but on the other hand, L-Arg can also be hydrolyzed by arginases to ornithine and urea; thus arginase competes with eNOS for the common substrate [121]. Arginase exists in two isoforms, Arg-I and Arg-II, encoded by two separate genes [131]. Arg- I is located mainly in the liver and participates in the last step of the urea cycle, converting L-Arg to L-ornithine and urea. Arg-II is scattered across different body tissues, most abundant in the kidney but also found in the endothelium, and functions independently of the urea cycle [132]. Both isoforms have been reported to be expressed in endothelial cells; however, their expression seems to be species and vascular bed–specific [133]. For example, Arg-I is barely detectable in HUVECs, but both arginase isoforms are present in human aortic endothelial cells (HAECs) [134]. As arginase and nitric oxide synthase compete for the same substrate, upregulation or activation of arginase can impair nitric oxide generation by eNOS. Excessive arginase expression or activity can result in eNOS uncoupling [135].

Hypoxia has been shown to be one of the factors that stimulate arginase. It was reported that hypoxia upregulates the expression and activity of Arg-II in human pulmonary microvascular endothelial cells (HPMECs), HUVECs, and BAECs, with concomitant reduction in eNOS activity and decrease in NO release [134,136,137]. Hypoxic upregulation of Arg-II has been shown to be mediated through HIF2-α in HPMEC [136], whereas in HUVECs, hypoxia as well as hypoxia mimetic DMOG, upregulated Arg-II in HIF-1α-dependent manner [138]. In HPMECs, hypoxia was reported to upregulate Arg-II through increased degradation of KLF15, which under normoxic conditions inhibit arginase transcription [139]. Furthermore, the cellular distribution of Arg-II was shown to be affected by hypoxia: Arg-II and eNOS proximity is increased in hypoxia, which allows arginase to successfully compete for the substrate [134]. HIF-2 has also been shown to be involved in the pathogenesis of hypoxic pulmonary hypertension by inducing the expression of Arg-I as well as Arg-II in the pulmonary endothelium, thus disrupting NO homeostasis [140]. Increased expression of Arg-II was reported to be responsible for impaired NO synthesis in endothelial cells of patients with PH [141].

The major fraction of plasmatic and cellular arginine in adult humans comes from physiological whole-body protein turnover [121]. Due to common post-translational modification, L-Arg residues within proteins can be methylated by a family of enzymes named protein arginine methyltransferases (PRMTs) [142]. The subsequent breakdown of such proteins results in the release of free methylated arginine derivatives: NGmonomethyl-L-arginine (L-NMMA), asymmetric dimethylarginine (ADMA), and symmetric dimethylarginine (SDMA) [143]. Methylarginies released into the cytosol pass into the plasma and can be taken up by other cells via y+ carriers. Both ADMA and L-NMMA, but not SDMA, are potent competitive inhibitors of nitric oxide synthases, with blood ADMA concentrations approximately 10-fold higher than L-NMMA. If taken up by endothelial cells, ADMA is the major endogenous inhibitor of eNOS, and disturbed L-Arg/ADMA ratio can lead to eNOS uncoupling [144]. Furthermore, ADMA and SDMA interfere with the cellular uptake of L-Arg by y+ carrier and thereby potentially reduce L-Arg uptake [145,146]. Increased ADMA levels in plasma have been correlated with endothelial dysfunction and are an independent risk factor for the development of systemic cardiovascular diseases [147].

About 20% of circulating ADMA is eliminated by kidneys, and the remaining 80% is metabolized by dimethylarginine dimethylaminohydrolases (DDAHs) that degrade ADMA to L-citrulline and dimethylamine (DMA). DDAHs are expressed in two isoforms. DDAH-1 is mainly detected in kidneys, liver, brain, and lungs but is also found in the endothelium, and it is believed to be responsible for the systemic elimination of circulating ADMA. DDAH-2 is expressed primarily in blood vessels (including endothelium), heart, placenta, and immune tissues, and its role in ADMA metabolism seems to be more local [148]. Diminished DDAH expression and activity have been linked to ADMA accumulation and endothelial dysfunction [149].

Hypoxia can affect ADMA generation and metabolism [150]. First, hypoxic conditions may increase protein L-Arg methylation. It was reported that the expression of one of the isoforms of protein arginine methyltransferases, PRMT2, is upregulated in the lungs of mice exposed to chronic hypoxia, resulting in increased protein methylation and elevated ADMA levels [151]. Thus, hypoxia may affect ADMA levels by increasing protein L-Arg methylation. Second, hypoxia can affect DDAH activity and ADMA metabolism. ADMA has been shown to accumulate in animal hypoxia models, as well as in human studies [152]. Higher ADMA but lower L-Arg serum levels were observed in patients with obstructive sleep apnea syndrome, in relation to healthy controls [153] and in patients with pulmonary hypertension [154]. In the chronic hypoxia-induced pulmonary hypertension model, increased ADMA levels were observed along with reduced DDAH-1 expression and activity [155]. In vitro studies have shown that 48h of hypoxic treatment led to diminished DDAH-1 expression and elevated ADMA levels in human pulmonary artery endothelial cells (HPAECs) [156]. On the other hand, exogenously added ADMA increased the stabilization of HIF-1α protein in HPAECs, further decreasing DDAH-1 expression and activating HIF-1α dependent pathways, resulting in the development of pulmonary hypertension phenotype. These results suggest that ADMA accumulation induced by hypoxia can be involved in the pathogenesis of pulmonary hypertension [156]. Iannone et al. demonstrated [157] that endothelium-specific upregulation of miR-21 by hypoxia is responsible for decreased DDAH-1 expression and ADMA accumulation, leading to the development of hypoxia-induced pulmonary hypertension. Increased miR-21 and diminished DDAH-1 expression were observed in hypoxic HPAECs, as well as in lung tissues from patients with idiopathic pulmonary arterial hypertension [157]. Some studies show that hypoxia can also negatively regulate DDAH-2. Arrigoni et al. [158] reported an approximately 90% reduction in DDAH2 protein, paralleled by ca. 70% decrease in DDAH activity in the lungs of neonatal piglets exposed to hypoxia, a porcine model of pulmonary hypertension. Reduced DDAH2 protein and mRNA, paralleled by elevated ADMA, were observed in patients with idiopathic pulmonary arterial hypertension and in pulmonary hypertensive rats [159].

**Figure 2:**
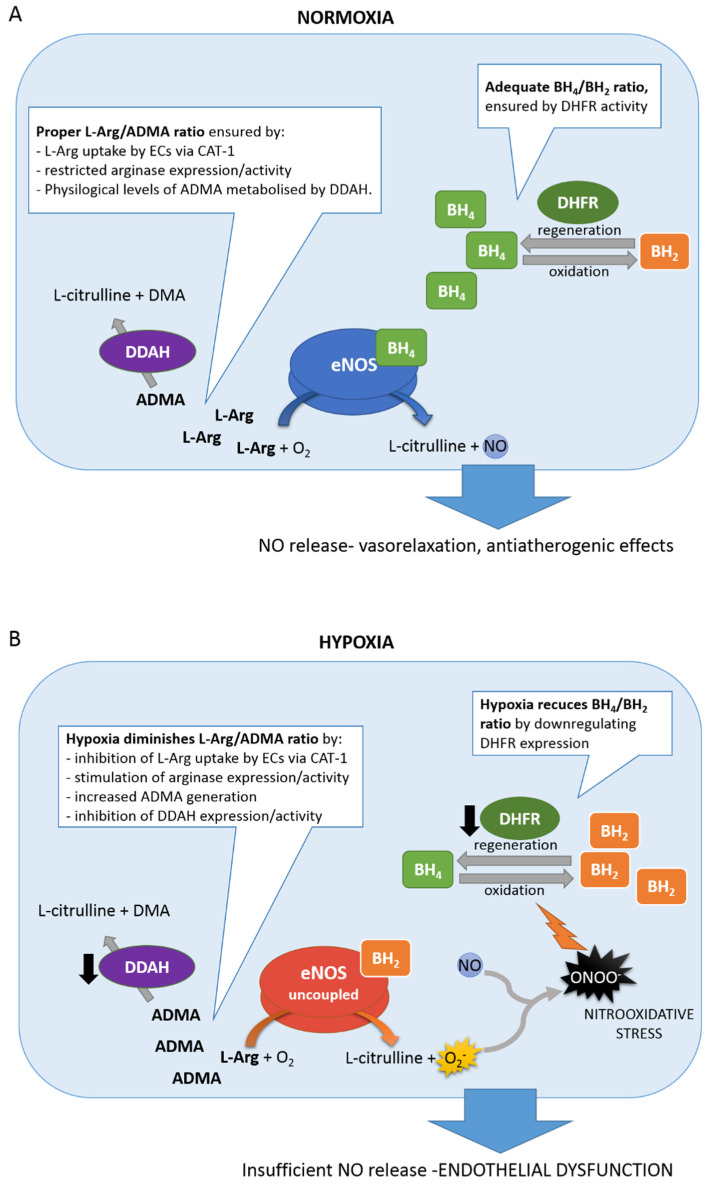
(**A**) In normoxic endothelial cells, eNOS in its “coupled” state produces appropriate amounts of NO, which protects against atherogenesis. (**B**) Under hypoxic conditions, DHFR expression and activity are diminished. ADMA accumulates as a result of increased PRMT activity and inhibition of DDAH expression and activity, while intercellular L-Arg levels are reduced due to inefficient transport and increased arginase activity. Decreased L-Arg/ADMA and BH_4_/BH_2_ ratios contribute to eNOS uncoupling and O_2_^−^ generation. O_2_^−^ reacts with NO (still produced in smaller quantities), leading to formation of ONOO^−^, which further oxidizes BH_4_, initiating the vicious cycle that results in oxidative stress. Reduced NO release (i.e., endothelial dysfunction) leads to atherogenesis.

As discussed above, hypoxia via disturbing the BH_4_/BH_2_ ratio or L-Arg/ADMA ratio can induce eNOS uncoupling, which in turn evokes oxidative stress, further intensifying eNOS uncoupling and leading to endothelial dysfunction (Figure 2).

## 7. Hypoxia, Oxidative Stress and Endothelial Inflammation

When discussing the influence of hypoxia on NO production in the endothelium, one should not ignore the accompanying oxidative stress and inflammation, as they are the basic mechanisms of atherosclerosis pathobiology, and hypoxia may trigger or intensify both these pathways.

It is well established that one of the mechanisms by which hypoxia induces tissue damage is the increased formation of reactive oxygen species (ROS). Endothelial cells respond to acute hypoxia by rapid and transient superoxide generation by the mitochondrial electron transport chain [160,161]. In addition, the activity or expression of NADPH oxidase can be increased in hypoxic conditions, augmenting overall ROS production [162,163]. The excess superoxide scavenges NO generated by eNOS in endothelial cells, thus limiting NO bioavailability. The resulting peroxynitrite oxidizes the BH_4_ cofactor, leading to eNOS uncoupling [164]. Furthermore, oxidative stress disturbs the balance between L-Arg and ADMA [165]. It was shown that ROS impair endothelial NO-mediated coronary microvessel dilation by upregulating arginase activity and reducing L-Arg availability [166]. Arginase expression and activity were also shown to be elevated in ROS exposed BAECs through the RhoA/ROCK pathway [167]. Thus, hypoxia through arginase upregulation and eNOS uncoupling can partly contribute to oxidative stress. It was shown that arginase inhibition reduced hypoxia-induced ROS formation [137]. In fact, oxidative stress is considered as the major contributor to eNOS uncoupling and endothelial dysfunction [59,168]. Hence, hypoxia not only reduces the bioavailability of nitric oxide directly (as discussed in previous sections) but also acts indirectly by inducing oxidative stress. Moreover, hypoxic response and oxidative stress are interrelated: mitochondria-derived ROS have been shown to mediate the hypoxic response by acting as signaling molecules that contribute to HIF-α stabilization, and hypoxic response is lost in cells depleted of mitochondrial DNA (ρ^0^ cells) [169,170].

In addition to oxidative stress, hypoxia can induce endothelial cell activation, a proinflammatory and procoagulant state of ECs, characterized by the expression of cell–surface adhesion molecules. ECs activation is driven by the NF-κB transcription factor, which is rapidly induced in response to a stimulus such as cytokines, bacterial or viral antigens, and stress signals, including hypoxia [171,172]. Upon activation, NF-κB induces the expression of proinflammatory genes, mainly cytokines (TNF-α, IL-1, IL-2, IL-6, IL-8, IL-18) and adhesion molecules (VCAM-1, ICAM-1, E-selectin) that mediate leukocyte rolling, adhesion, and transendothelial migration, and initiate inflammatory cascade and atherogenesis [173,174]. Hypoxia has been shown to induce NF-κB via mechanisms reviewed elsewhere [175], and the effects of NF-κB signaling were observed in vivo and in vitro. Both OSA patients and people with mountain sickness have been reported to present elevated levels of circulating proinflammatory cytokines linked to NF-κB activation [176,177,178]. Expression of ECs activation markers has also been demonstrated in cerebral microvessels of rats exposed to hypoxia [55]. Low oxygen tension, as well as hypoxia mimetic DMOG, upregulated ICAM-1 expression in HUVECs [138]. Similarly, overexpression of HIF-1α and HIF-2α in endothelial cells elevated their surface expression of VCAM-1 and ICAM-1, and consequently increased their adhesion and migration capacity [179].

Atherosclerosis is a chronic inflammatory state, initiated with endothelial cell activation. Endothelial activation, in turn, is associated with endothelial dysfunction since reduced NO bioavailability stimulates endothelial activation. De Caterina et al. [180] showed that inhibition of eNOS with L-NAME resulted in induction of VCAM-1 expression, a marker of ECs activation and inflammation. On the other hand, NO donors reduced the expression of adhesion molecules and proinflammatory cytokines through inhibition of NF-κB [180]. Conversely, ECs activation was shown to contribute to endothelial dysfunction by inhibiting eNOS expression via decreasing eNOS promoter activity, as well as its mRNA half-life due to miR-155 induction [101,181,182]. In addition, hypoxia and the HIF-1α pathway are associated with the accumulation of advanced glycation end products (AGEs) and increased RAGE (AGEs receptor) signaling, which in turn also contributes to oxidative stress and endothelial dysfunction [183,184]. The role of RAGE activation in diseases with ischemic background, such as atherosclerosis, peripheral artery disease, or cancer, was well established in both human cell studies and animal models [185,186,187].

**Figure 3:**
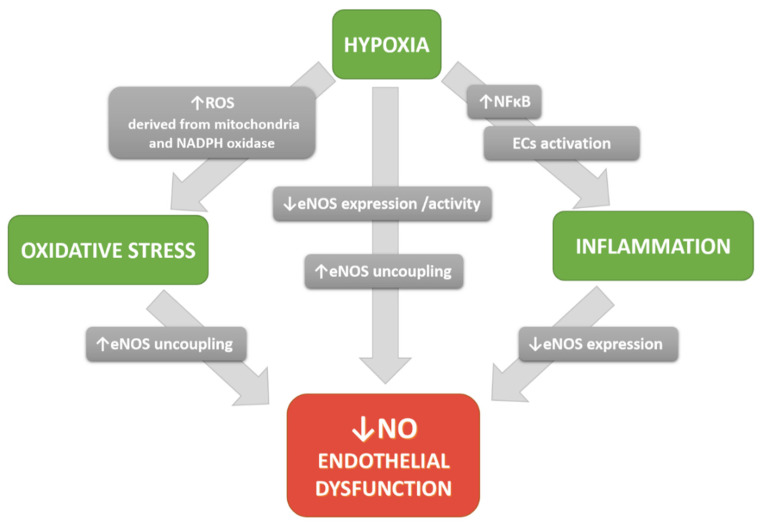
Scheme illustrating the influence of hypoxia on NO bioavailability—major pathways regulating NO bioavailability in the endothelium and the interrelationship between them.

Hypoxia elicits ECs activation and inflammation but, on the other hand, inflammatory diseases are frequently characterized by tissue hypoxia. It has been demonstrated that atherosclerotic lesions contain regions of severe hypoxia [76,77]. On the other hand, HIF-α accumulation can also be observed in normoxia. Interestingly, NF-κB upregulates HIF-1α in response to elevated ROS, resulting in the induction of HIF-dependent target genes [188]. Thus, the hypoxic response, oxidative stress, and inflammation are interrelated and overlapping mechanisms involved in the pathological reduction in NO synthesis and availability, leading to endothelial dysfunction (Figure 3).

## 8. Concluding Remarks

In conclusion, hypoxia affects the nitric oxide synthesis pathway through several convergent and interdependent mechanisms. Hypoxic signaling diminishes eNOS expression and activity and, most importantly, evokes eNOS uncoupling via disturbing the activity or expression of BH_4_ and L-Arg/ADMA-related enzymes. Uncoupled eNOS is an important source of free radicals that disrupts redox balance and evokes oxidative stress. Simultaneously, hypoxia contributes to the impairment of the NO pathway by increasing free radicals derived from the mitochondrial respiratory chain and NADPH oxidase and by stimulating endothelial activation. Together, these disturbances contribute toward the reduction in nitric oxide bioavailability and the development of endothelial dysfunction, which underlies the pathophysiology of cardiovascular diseases. Understanding the interdependence between these seemingly different mechanisms can help to develop therapeutic strategies for the prevention of atherosclerosis.
